# Contribution of Hydrogen Bonds to Paper Strength Properties

**DOI:** 10.1371/journal.pone.0155809

**Published:** 2016-05-26

**Authors:** Piotr Przybysz, Marcin Dubowik, Marta Anna Kucner, Kazimierz Przybysz, Kamila Przybysz Buzała

**Affiliations:** 1 Institute of Papermaking and Printing Technology, Lodz University of Technology, Wolczanska str. 223, 90–924 Lodz, Poland; 2 Faculty of Process and Environmental Engineering, Lodz University of Technology, Wolczanska 213, 90–924 Lodz, Poland; Monash University, AUSTRALIA

## Abstract

The objective of this work was to investigate the influence of hydrogen bonds between fibres on static and dynamic strength properties of paper. A commercial bleached pinewood kraft pulp was soaked in water, refined in a PFI, and used to form paper webs in different solvents, such as water, methanol, ethanol, n-propanol and n-butanol, to determine the effect of their dipole moment on static and dynamic strength properties of resulting paper sheets. Paper which was formed in water, being the solvent of the highest dipole moment among the tested ones, showed the highest breaking length and tear resistance. When paper webs were formed in n-butanol, which was the least polar among the solvents, these parameters were reduced by around 75%. These results provide evidence of the importance of water in paper web formation and strong impact of hydrogen bonds between fibres on strength properties of paper.

## Introduction

Paper is a versatile material used in everyday life and various branches of industry and economy. Global production and consumption of paper products amount to 400 million metric tons and are continually growing [1,2]. Apart from functionality and relatively low price, paper products belong to environmentally friendly materials, derived from fibrous crops, mainly wood, and recycled streams such as recovered paper [3,4].

Most important factors deciding of paper strength characteristics are properties of fibres forming its structure (among others fibres length and strength) as well as fibre-to-fibre interactions [5]. Hydrogen bonds and friction forces between fibres in paper are the dominating of them [6,7,8].

Polar water molecules play the key role in formation of hydrogen bonds between fibres in paper [9,10]. In papermaking, all technological operations take place in water, whose consumption amounts to ca. 5 to 15 m^3^ per ton of paper produced [2,11,12]. Because of the relatively low price, water is used as an inexpensive transportation (slurry pumping), cooling, sealing and washing medium. Relatively less attention is paid to the role of water as a chemical medium taking part in successive steps of papermaking process, including formation of hydrogen bonds. This article presents results of the study on the influence of water and other solvents (alcohols of different molecular weights and dipole moments) on the energy of hydrogen bonds between fibres and paper strength properties [13,14].

## The Nature and Significance of Paper Web Formation Process

Web formation from a diluted papermaking slurry (dry weight of about 0.5%) takes place on a moving wire screen. Dewatering of this slurry results in the merger of fibres and fines into a web of defined structure and dryness of 15 to 25%. Further dewatering of the web via pressing and drying processes, yields the sheet of paper with defined properties [8,15,16]. Currently applied paper machines can produce paper sheets of the width up to 12 m at the rate of about 30 m/s (ca. 110 km/h) and yield of ca. 4500 t/d.

**Fig 1:**
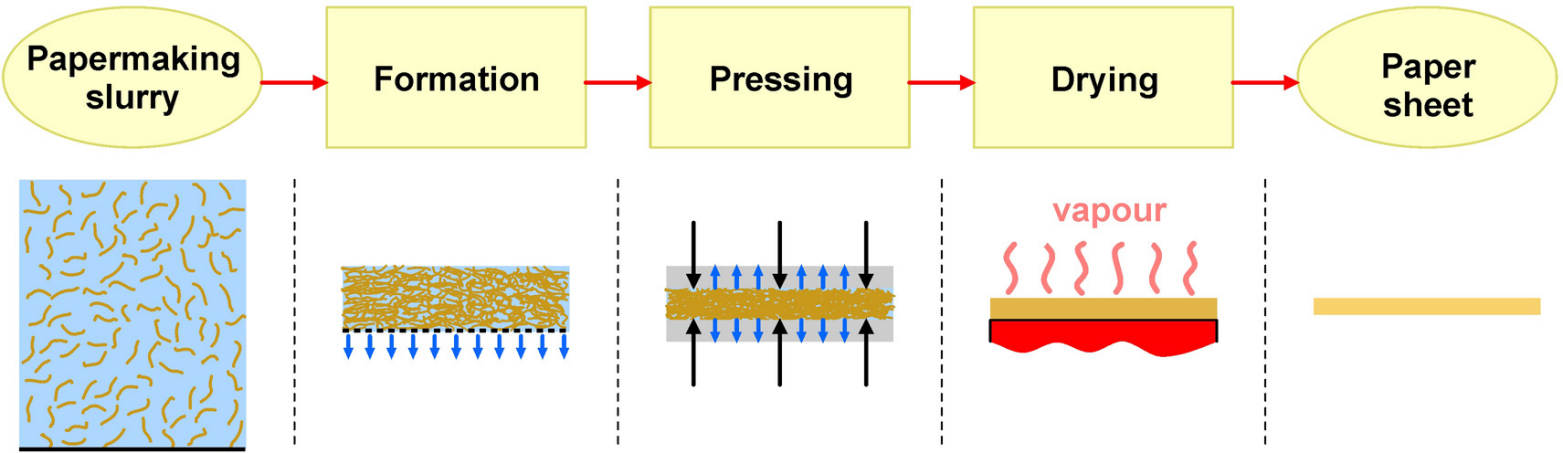
Successive steps of paper sheet formation.

The consolidation of paper sheet and development of its structure is a result of dewatering of papermaking slurry when paper web is passed through successive sections of a paper machine (Fig 1).

Paper web of defined structure and dry weight of ca. 20% (Fig 1B) is formed through gravitational and/or vacuum dewatering of a slurry containing single fibres and their aggregates (flocs) (Fig 1A) in a wire screen section of the paper machine [7,17].

Further dewatering to ca. 40% d.w. takes place in a press section. It results in paper sheet consolidation. Free water is practically removed from the spaces between fibres in this stage (remains only water contained inside the fibres) [7,18]. Fibres forming the web are separated only by the layer of water adsorbed on their surfaces [19,20,21,22] (Fig 1C).

Water contained inside the fibres and adsorbed on their surfaces is removed during the drying process (Fig 1D). The removal of water from the interior of fibres causes their shrinkage and results in the shrinkage of the sheet and modification of its structure. The removal of adsorbed water causes an increase in the strength of interactions between fibres in paper [23,24].

Hydrogen bonds and friction forces between fibres are the principal forces deciding of the strength of interactions between fibres in the structure of paper [2,7,8,25]. These interactions become noticeable when external forces act on paper, and have a strong impact on its strength properties [26,27].

Hydrogen bonds are formed mainly between hydroxyl groups of cellulose and hemicellulose fibres that are contained in papermaking pulps. The displacement of the negative charge towards oxygen takes place in these groups as a result of the high electronegativity of oxygen. Hydroxyl groups (–OH) behave like dipoles with the negative charge shifted to oxygen and the positive charge close to hydrogen [28].

The relatively strong noncovalent interactions, known as hydrogen bonds, are formed when the distance between two hydroxyl groups is around 0.26 nm. The energy of these bonds reaches 62.8 kJ/mol [29].

The presence of water molecules is necessary for formation of hydrogen bonds between fibres in paper. Oxygen atom and two atoms of hydrogen form a triangle with an apex angle of 105°. Water molecule due to the irregular electric charge arrangement is in general presented as a dipole with a dipole moment of 1.85 D and the negative pole on the oxygen atom and the positive one close to the hydrogen atoms.

**Fig 2:**
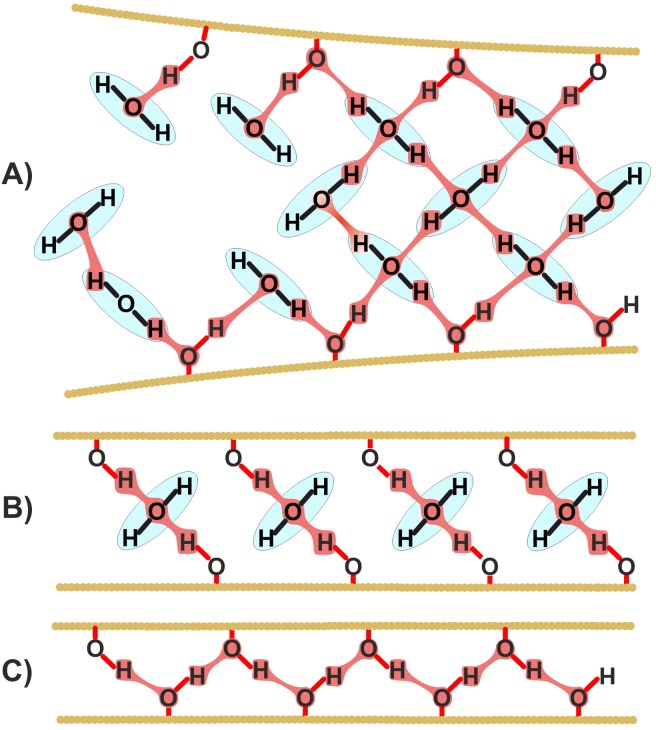
Successive steps of hydrogen bonds formation between fibres in paper. A) bonding via a multimolecular water layer, B) bonding via a monomolecular layer of water, C) hydrogen bonds.

Paper web formation and drying result in paper structure consolidation [26,30,31]. Water molecules are either removed by mechanical pressing or evaporated, and fibres come closer (Fig 2). The last phase of this process when fibres are separated only by a few layers of water results in formation of water bridges between hydroxyl groups of contiguous fibres (Fig 2A and 2B). Then these bridges turn into hydrogen bonds (Fig 2C).

The polar character of water molecules is a very important factor, enabling formation of multiple hydrogen bonds between cellulose fibres in paper structure.

Although water is regarded as an easily available and inexpensive solvent, in which all papermaking operations take place, the importance of water as a crucial factor influencing mechanism and outcomes of the papermaking process is usually underestimated in scientific literature. Better understanding of the role of water in paper structure formation may have both theoretical meaning and practical importance. The energy of fibre-to-fibre bonds in paper is a factor deciding of its strength properties [32].

Literature data [6,10] suggest that the energy of fibre-to-fibre bonds in paper is approximately a sum of the energy of hydrogen bonds and the energy of friction forces between fibres when paper sheets are torn. The replacement of polar water molecules in papermaking pulps with solvents of lower polarity may reduce the energy of hydrogen bonds and very slightly affect these properties of fibres (surface roughness, dimensions), which decide of the energy of friction forces between them. Therefore, the replacement of water by alcohols of different polarity may enable estimation of: (i) the influence of fibre-to-fibre bonds in paper on its strength properties and (ii) contribution of the energies of hydrogen bonds and friction to the total energy of interactions occurring between fibres in paper.

## Materials and Methods

To determine the role of water in formation of hydrogen bonds and their influence on paper strength properties, water was replaced by four solvents with increasing molecular weight and decreasing polarity (Table 1). The alcohols shown in Table 1 were selected as water replacements because the hydroxyl groups (-OH) are necessary for hydrogen bonds formation.

**Table 1:** Selected properties of applied solvents [33].

Liquid	Chemical formula	Molecular mass	Electric permittivity	Dipole moment
-	-	μ	F/m	D
**water**	H-OH	18	80	1.85
**methanol**	CH_3_-OH	32	33	1.7
**ethanol**	CH_3_-CH_2_-OH	46	24	1.69
**n-propanol**	CH_3_-CH_2_-CH_2_-OH	60	20	1.68
**n -butanol**	CH_3_-CH_2_-CH_2_-CH_2_-OH	74	18	1.66

### Cellulosic pulp

A typical air dried bleached pine kraft pulp was used in the study.

The following parameters were measured for the unbeaten pulp:

- dryness– 96.0% according to ISO 638:2008, the standard deviation of results of four measurements was 0.1%
- lignin content–Kappa number of 0 according to ISO 302:2004, the measurements were conducted in triplicate and the standard deviation was 0.1
- swelling in water (WRV–Water Retention Value)—93% according to ISO 23714:2014, the standard deviation of results of four measurements was 0.6%
- average fibre length– 2.30 mm, measured using a Kajaani FS-200 device, the measurements were performed in triplicate and the standard deviation was 0.01 mm.

### Pulp beating

Pulp samples (22.5 g d.w.) were soaked in demineralized water overnight and then defiberized in a laboratory appliance according to ISO 5263–1:2006. The defiberized samples were concentrated up to 10% d.w. and beaten in a PFI mill under standard conditions (according to ISO 5264–2:2011).

The results of preliminary beating tests showed that the standard Schopper-Riegler number of SR-30 was reached within 3 min. Paper produced from this pulp was characterized by the highest static strength properties.

Pulp properties after beating were as follows:

✓ Schopper-Riegler freeness–SR-30 according to ISO 5267–1:2002, the measurements were performed in triplicate and their results were the same,
✓ WRV– 201.0% (according to ISO 23714:2014), the standard deviation of results of four measurements was 0.8%,
✓ average weighted fibre length– 1.76 mm, the measurements were performed in triplicate and the standard deviation was 0.01 mm,
✓ fines content– 13.2% d.w. measured with a H-S Lorentzen-Wettre apparatus, the measurements were carried out in triplicate and the standard deviation was 0.5%.

### Properties of paper

Handsheets were produced with the use of a standard laboratory Rapid-Köthen sheet former, according to PN-EN ISO 5269–2:2007.

The following paper properties were measured after conditioning in the standard atmosphere, according to ISO 187:1990 (at air relative humidity φ of 50% and temperature of 23°C):

- grammage– 75 g/m^2^ (according to ISO 536:2012), only paper sheets of grammage between 74 to 76 g/m^2^ were accepted for further investigation,
- bulk density (according to PN-EN ISO 534:2012), twelve measurements were made,
- breaking length, elongation and breaking energy (according to PN-EN ISO 1924–2:2010), twelve measurements were made,
- tear resistance (according to PN-EN ISO 1974:2012) twelve measurements were made,
- burst (according to PN-EN ISO 2758:2005), 12 measurements were made,
- brightness (according to Tappi T452), 6 measurements were made,
- diffuse opacity (according to TAPPI T519), 6 measurements were made.

Formation of sheets in selected alcohols was carried out with the use of the Rapid-Köthen sheet former (according to PN-EN ISO 5269–2:2007) after water removal from pulp samples and its replacement by one of the alcohols.

Water was separated from pulp samples through washing with the selected alcohols. When the sheets were formed in methanol and ethanol (anhydrous), samples of pulp, which were subjected to beating in the PFI mill, were placed on a vacuum filter and washed with 500 ml of alcohol (added in four approximately 125 ml portions). Then the samples of pulp were placed in a hermetic vessel and each of them was mixed with 400 ml of the alcohol and incubated for 1 hour in a water bath at the temperature of 50°C to accelerate the displacement of water. This operation was repeated twice.

When the sheets were formed in propanol, pulp samples were at first washed with methanol and then twice with propanol, as described above. The samples of pulp used to form sheets in n-butanol were successively treated with methanol, n-propanol and at the end with n-butanol, as described above.

Pulp samples, which contained the alcohols instead of water, were transferred to the defiberizer and each of them was mixed with the same alcohol to obtain the slurry with the volume of 7 dm^3^, which was subjected to defiberization. Then samples containing 2.34 g of the pulp were taken to form 75 g/m^2^ laboratory test sheets, according to ISO 5269–2:2007, using 5 dm^3^ of the alcohol.

The following properties of the sheets formed were measured after conditioning in standard conditions (according to PN-EN 20187:2000):

- grammage (according to ISO 536:2012),
- calliper and bulk density (according to ISO 534:2012),
- breaking length, elongation and breaking energy (according to ISO 1924–2:2010),
- tear resistance (according to ISO 1974:2012).
- burst (according to PN-EN ISO 2758:2005)
- brightness (according to Tappi T452),
- diffuse opacity (according to TAPPI T519),

## Results and Discussion

The replacement of water with the four tested alcohols changed neither the strength nor the length of fibres contained in the papermaking pulps, which were prepared and used to form paper sheets in this study. Therefore, the change in the tensile and structural properties of paper (Tables 2 and 3), caused by the replacement of water with each of the alcohols, was ascribed to the reduced energy of hydrogen bonds between fibres.

**Table 2:** WRV of pulp, bulk density and optical properties of paper sheets formed in water and different alcohols.

Forming liquid	WRV [%]		Bulk density [g/cm^3^]		Brightness [%]		Diffuse opacity [%]	
	Mean value	SD*	Mean value	SD	Mean value	SD	Mean value	SD
Water	201.45	0.63	0.6653	0.0040	102.42	0.01	54.05	0.21
Methanol	153.32	0.42	0.6095	0.0014	98.65	0.09	63.71	0.20
Ethanol	137.05	0.35	0.6076	0.0052	96.49	0.09	65.14	0.22
Propanol	122.32	0.48	0.5883	0.0020	92.13	0.04	68.37	0.02
n-butanol	99.90	0.28	0.05798	0.0016	102.42	0.01	53.14	0.05

* SD stands for standard deviation.

**Table 3:** Tensile properties of laboratory paper sheets formed in water and different alcohols.

Forming liquid	Braking length [m]		Tear resistance [mN]		Burst [kPa]		Breaking energy [J]	
	Mean value	SD*	Mean value	SD	Mean value	SD	Mean value	SD
Water	8020	110	581	22	481	19	0.169	0.013
Methanol	3940	340	400	37	241	13	0.104	0.012
Ethanol	3690	230	379	29	230	23	0.102	0.014
Propanol	3020	250	304	40	225	16	0.096	0.014
n-butanol	1800	240	177	19	216	19	0.055	0.011

* SD stands for standard deviation.

The sheets formed in water had the highest breaking length, of 8 020 m. This parameter was reduced when the sheets were formed in the alcohols and was negatively correlated with their molecular weight. The values of breaking length were positively correlated with the polarity of the alcohols and varied between 3 940 m for methanol and 1 800 m for n-butanol.

It was found that the decrease in the dipole moment of alcohol resulted in the reduced paper breaking length. When the web was formed in butanol, which had the lowest dipole moment of 1.66 D, the breaking length was only about 1 800 m, rendering this paper useless for the majority of typical applications.

The decrease in the dipole moment from 1.85 D (for water) to 1.66 D (for butanol) caused an about 78% decrease in the paper breaking length, from 8 020 m to 1 800 m, that may be ascribed to the decrease in the energy of hydrogen bonds between fibres.

The same situation was observed for changes in other static tensile properties like burst and breaking energy. Burst for paper sheets formed in n-butanol was reduced from 481 to 216 kPa (by about 55%) and breaking energy from 0.169 to 0.55 J (by about 67%).

This suggests that the energy of hydrogen bonds decides of paper static strength properties and the impact of the energy of friction forces on the paper breaking length is much lower. The obtained results suggest that in up to about 70% the tensile properties are related to the hydrogen bonds between fibres and in around 30% are caused by friction forces between fibres.

Tear resistance is a measure of dynamic strength properties of paper, which are positively correlated with the length and strength of fibres and depend also on the energy of fibre-to-fibre interactions. The paper sheets were formed from the same pulp so the length and strength of fibres were the same in all cases. Usage of different solvents in web formation decided of the energy of bonds and tear resistance of paper.

Like the breaking length also the tear resistance of paper was the highest, of 581 mN, when the webs were formed in water and decreased with the increase in molecular weight and the decrease in polarity of alcohols. It reached 400 mN for methanol and only 177 mN for n-butanol.

When the dipole moment of the alcohol (butanol) equalled 1.66 D, the tear resistance of paper was only about 177 mN, which means that this paper was useless for the majority of typical applications.

These data clearly demonstrate that the decrease in the dipole moment of the solvent used for web formation from 1.85 D (for water) to 1.66 D (for butanol) caused a decrease in the paper tear resistance from 581 mN to 177 mN, it means by ca. 70%. This was ascribed to the reduced energy of hydrogen bonds between fibres. Thus also dynamic strength properties of paper depended mainly on the energy of hydrogen bonds between fibres while the impact of the energy of friction forces on the paper tear resistance was much lower.

**Fig 3:**
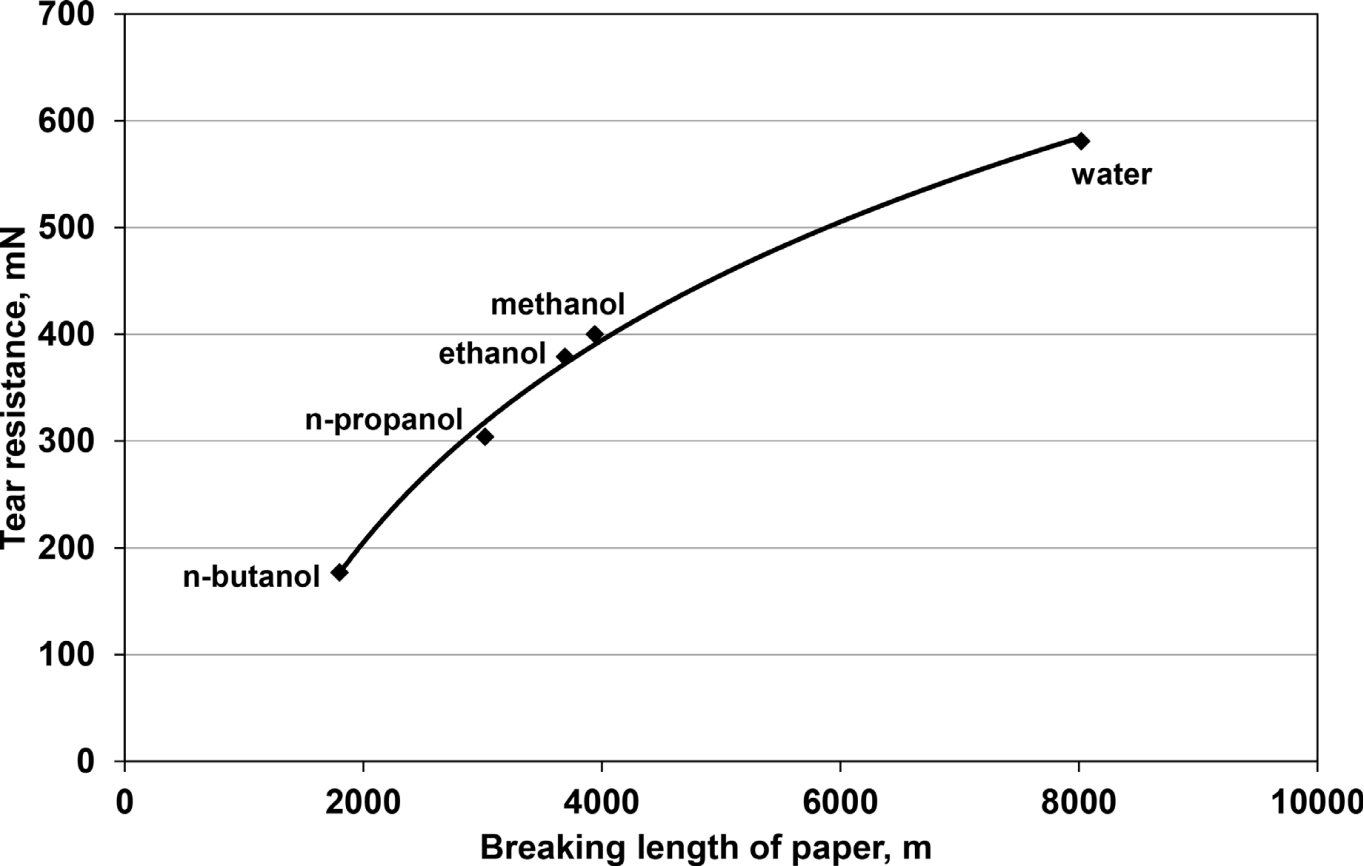
The interplay between the tear resistance and breaking length of paper formed in water and selected alcohols.

The interplay between the breaking length and tear resistance of paper sheets obtained in this study is presented in Fig 3.

The sheets formed in water were characterized by the highest breaking length and tear resistance, of 8 020 m and 581 mN, respectively. The values of these two parameters decreased with the drop in polarity and the rise in molecular weight of the solvent. They were relatively high for methanol, of 3 940 m and 410 mN, respectively, and much lower for n-butanol, of 1 800 m and 177 mN, respectively. Thus, paper prepared in the latter solvent, with polarity of 1.66 D, exhibited such low properties so that it could not find any potential application.

Because all paper sheets formed in this study contained fibres of the same length and strength, the principal factor deciding of the energy of interactions between fibres, which in turn determines the breaking length and tear resistance of paper, was the dipole moment of the solvents used for web formation.

## Conclusions

The results of this study enabled to determine the effect of the polarity of solvents used for web formation on paper strength properties, and to evaluate the participation of hydrogen bonds in formation of paper properties.

1. The decrease in the dipole moment of the solvent used for web formation from 1.85 D (for water) to 1.66 D (for butanol) caused the decrease in the paper breaking length (from 8 020 m to 1 800 m), burst (from 481 to 216 kPa), and breaking energy (from 0.169 to 0.055 J). Reduction of these parameters by 55% to 70% may be ascribed to the decrease in the energy of hydrogen bonds between fibres. It can therefore be concluded that hydrogen bonds are responsible for more than half of the static strength properties of the paper.
2. Also the paper tear resistance was positively correlated with the dipole moment of the solvent used for web formation, and was reduced from 581 mN to 177 mN when this parameter was lowered from 1.85 D (for water) to 1.66 D (for butanol). Similarly to the static tensile properties, also dynamic properties were deteriorated. Since the average fibre length in each case was the same, the changes observed reflected the lower bonding energy between the fibres.
3. The obtained results show that the hydrogen bonds are much more important for paper strength properties than the friction forces between fibres. Hydrogen bonds are responsible for at least 60% of tensile properties of paper.
4. The analysis of standard deviation of breaking length of paper formed in water and selected alcohols shows that results for breaking length of paper formed in water were more convergent. Standard deviation of breaking length of paper formed in water was two–three fold lower compared to paper formed in alcohols. This suggests that paper formed in water had more uniform structure than paper formed in alcohols. However, results concerning other tensile properties did not support this observation.
5. Formation of paper in water and selected alcohols significantly affected optical properties of paper. The values of ISO brightness ranged from 102.42% for water and butanol to 92.13% for propanol. The changes in diffuse opacity that were also observed, were negatively correlated with the bulk density of paper.
